# Quantifying the Impact of COVID-19 on Cancer Patients: A Technical Report of Patient Experience During the COVID-19 Pandemic at a High-volume Radiation Oncology Proton Center in New York City

**DOI:** 10.7759/cureus.7873

**Published:** 2020-04-28

**Authors:** Robert H Press, Shaakir Hasan, Arpit M Chhabra, J. Isabelle Choi, Charles B Simone

**Affiliations:** 1 Radiation Oncology, New York Proton Center, New York, USA

**Keywords:** covid-19, radiation oncology, patient safety, new york, covid-19 in cancer patients, corona virus, proton therapy

## Abstract

The COVID-19 pandemic has rapidly spread across the world and now affects more people within the United States than any other country. New York City has emerged as the epicenter of the outbreak in the United States. Both locally and across the country, there is great concern in our ability to deliver appropriate medical care during this time. Radiation therapy is another essential clinical service that cannot afford to suffer prolonged delays without compromising patient outcomes. Early action and guidance are therefore critical to minimize transmission events and ensure safe and timely delivery of radiation therapy. The New York Proton Center (NYPC) is a high-volume free-standing multi-institutional proton center located in Manhattan. The purpose of this report is to describe the institutional patient experience and quantify the impact of treatment delays and interruptions over the first month of the COVID-19 outbreak. We also quantify the incidence of COVID-19 positive patients on census and provide guidance on proactive institutional policies to mitigate patient risk.

## Introduction

We commend Dinh et al. for their early and informative work describing the radiation therapy policy and procedural changes of the University of Washington (UW) in the Seattle-Puget Sound region during the initial stages of the U.S. COVID-19 outbreak [1]. Their proactive policies provided vital pre-emptive guidance to our department and radiation oncology departments across the country. After Seattle, New York City became one of the next major cities to be affected and has quickly emerged as the epicenter of the COVID-19 pandemic in the United States, with 76,876 confirmed cases as of April 7, 2020. 

The New York Proton Center (NYPC) is a freestanding facility located in Manhattan. Currently, five NYPC-employed radiation oncologists and 19 partner radiation oncologists from three consortium institutions (Memorial Sloan Kettering Cancer Center, Montefiore Health System, and Mount Sinai Health System) treat 80-85 patients/day. Since the first reported COVID-19 case in New York on March 1, 2020, the center has faced many challenges maintaining safe delivery of patient care. As the impact on radiation oncology patients of the COVID-19 pandemic is yet to be quantified, we describe some of our challenges and institutional policies aimed at mitigating the spread of COVID-19 amongst our patients and detail the proportion of patients affected during the initial one-month interval.

## Technical report

Similar to the UW, we instituted policies to minimize risks to patients, including providing patient education materials; completing daily symptom screenings of patients and close contacts; implementing rigorous sanitization measures; deferring treatment of indolent diseases; instituting telemedicine appointments, virtual meetings, restricting visitors, spacing-out treatment times, and closing patient waiting rooms. Patients in subacute care or nursing facilities were no longer eligible for treatment until discharged and we utilized hypofractionation to shorten treatment schedules per expert-consensus, when feasible [2-5]. Lastly, we began prospectively monitoring each patient on treatment and those expected to initiate treatment for new symptoms, date of onset, possible sick contacts, and COVID-19 test results. 

Based on increasing personnel losses due to illness and the need to optimize safety for all of our patients and staff, we deferred treatment of COVID-19-positive patients. By March 19, 2020, we instituted a new patient policy requiring symptomatic patients to obtain a negative COVID-19 test before treatment could resume/initiate, and COVID-19-positive patients must be serologically cleared prior to resuming. Patients with high-risk exposure are immediately quarantined and must either remain asymptomatic for a minimum of 10 days prior to treatment (i.e., two standard deviations of the average incubation period) and/or obtain a negative COVID-19 test, if available. Treatment starts are delayed to accommodate these requirements unless urgent circumstances are present, determined on a case-by-case basis.

Institutional Review Board approval was obtained for the evaluation of the following patient-level data. From March 1, 2020 to March 31, 2020, 137 patients received, or underwent simulation to receive, radiotherapy at NYPC. Of these 137 patients, 15 (11%) were monitored for concerning symptoms or high-risk exposures (Table 1). Alterations in treatment plans due to COVID-19 workup were necessary in 11 (8%) patients. The remaining four (3%) patients were monitored due to high-risk exposures (e.g., same household family member COVID-19 positive) but adequately evaluated and cleared through short-term quarantine, symptom evaluation, and/or negative COVID testing without impacting their treatment course. Of the 11 affected patients, seven were cleared and rescheduled for treatment, resulting in a median delay of seven days (range 2-32 days). Four patients, however, are indefinitely delayed or have stopped treatment entirely, including three confirmed COVID-19 infections in total. Of these three, one patient expired due to COVID-19 illness and one has initiated comfort care measures.

**Table 1:** Patient characteristics. CTX, chemotherapy; HN, head and neck; COVID, Coronavirus disease 2019; GI, gastrointestinal

Patient	Age (years)	Site	Concurrent CTX	On-treatment	Symptoms/Risk factors	Outcome	Days missed or delayed
1	81	HN	No	No	Asymptomatic Elderly	Quarantine delayed treatment start	19 days
2	86	HN	No	No	Fever, headache	COVID+ admitted to hospital, patient expired	Indefinitely
3	57	HN	No	No	Fever	COVID- delayed treatment start	7 days
4	59	HN	No	No	Cough, dyspnea, sore throat	COVID indeterminate quarantined 14 days without symptoms, plan to proceed with treatment	0 days
5	66	HN	Yes	Yes	Fever, cough	COVID- treatment interrupted	5 days
6	85	HN	No	Yes	Fever, cough, dyspnea	COVID+ admitted to hospital, comfort care measures initiated	Indefinitely
7	65	HN	Yes	Yes	Fever, cough	COVID- admitted for 4 days, treated for pneumonia	4 days
8	6	Brain	Yes	No	Asymptomatic indirect exposure	Quarantine resolved before treatment start	0 days
9	7	Brain	No	No	Fever admitted due to other medical reasons	Family refused COVID testing delayed until discharged	2 days
10	70	Brain	Yes	No	Asymptomatic indirect exposure	Quarantine	0 days
11	1	Sarcoma	Yes	No	Asymptomatic parents COVID+	Quarantine delayed treatment start	32 days
12	20	Sarcoma	Yes	Yes	Asymptomatic father is COVID+	Deferred COVID testing treatment interrupted	Indefinitely (ended early)
13	67	Thoracic	Yes	Yes	Cough, dyspnea	COVID+ admitted to hospital, treatment interrupted	Indefinitely
14	46	Breast	No	Yes	Asymptomatic indirect exposure	Quarantine	0 days
15	67	GI	No	No	Asymptomatic wife is COVID+	Quarantine delayed treatment start	20 days

The majority of patients who required monitoring had not yet started treatment (9/15, 60%). The most common scenario was the development of new symptoms prior to simulation. Of these patients, all but one has since been cleared and rescheduled, resulting in a median delay of 4.5 days (range 0-32 days). The one patient not yet cleared was confirmed COVID-19 positive and treatment remains on hold. Of the six patients on-treatment requiring further evaluation (40%), five suffered treatment interruptions, three of whom are now rescheduled to resume after a median delay of four days (range 0-5 days). Three remain indefinitely delayed, including two who were confirmed COVID-19 positive and one who terminated treatment early with two fractions remaining.

## Discussion

Given the proportion of patients who developed symptoms prior to initiating treatment, our experience supports the recommendation for rigorous screening in advance of treatment initiation as well as between appointments (e.g., when simulation and treatment start are >1 week apart). In addition, with increasing reports of asymptomatic transmission, routine inspection of lung imaging on simulation and cone-beam CT images is strongly encouraged [6-7]. Patients on-treatment were less commonly affected, which may be due to greater precautions to self-quarantine. However, on-treatment patients may also be more difficult to assess given ongoing treatment-related side effects. For example, some of the most common COVID-19 symptoms occur frequently among cancer patients receiving radiotherapy, including sore throat, cough, shortness of breath, and fatigue. Given the difficulty assessing patients on-treatment for COVID-19 related symptoms, we recommend close scrutiny of any new or ambiguous complaints and a low-threshold for viral testing.

We also have an average of five to seven pediatric patients on-treatment requiring daily sedation with anesthesia. UW’s anesthesia policy included airborne precautions due to reports of aerosol transmission [8]. NYPC instituted a policy on March 30, 2020 requiring COVID-19 testing before simulation, again prior to the first fraction, and weekly thereafter while on-treatment to further maximize safety. For patient comfort, the weekly diagnostic nasal swabs are obtained while the patient is under general anesthesia, typically after treatment is completed each Friday to allow test results to return before the subsequent treatment on Monday. 

Lastly, we assessed the utilization and sanitization procedure of our respiratory motion management system, SDX (DYN’R, Miami, FL). Similar to UW and Thomas Jefferson University, we developed a new and stringent disinfecting protocol and encouraged the use of abdominal compression and proton repainting techniques over breath-hold whenever clinically possible [9-10]. NYPC now requires manufacturer-recommended monthly full-system cleaning after every patient use which typically takes 10 min to perform. Prior to the COVID-19 outbreak, NYPC treated on average four SDX cases per day. Over the last two weeks prior to publication, only two patients remain on treatment using SDX and no new cases are currently under treatment planning. 

## Conclusions

Despite the early implementation of measures described by the UW radiation oncology department as well as our own highly conservative policies, NYPC still had 11% of patients affected, including 3% confirmed positive for COVID-19 within the first month and one patient death. All of the delays at NYPC occurred in the second half of the month, suggesting other cities should expect and prepare for an acceleration of patient events near their projected regional pandemic peak. While most interruptions were of short duration and did not appear clinically meaningful, implementation of these strict policies likely mitigated further patient exposure and more significant treatment delays. We plan to continue to enforce and reassess our policies throughout the pandemic to balance patient safety and optimal treatment delivery.
